# Presence of L1014F Knockdown-Resistance Mutation in *Anopheles gambiae s.s.* From São Tomé and Príncipe

**DOI:** 10.3389/fcimb.2021.633905

**Published:** 2021-07-07

**Authors:** Hongying Zhang, Mingqiang Li, Ruixiang Tan, Changsheng Deng, Bo Huang, Zhibin Wu, Shaoqing Zheng, Wenfeng Guo, Fei Tuo, Yueming Yuan, Carlos Alberto Bandeira, D’almeida Herodes Rompão, Qin Xu, Jianping Song, Qi Wang

**Affiliations:** ^1^ Artemisinin Research Center, Guangzhou University of Chinese Medicine, Guangzhou, China; ^2^ Science and Technology Institute, Guangzhou University of Chinese Medicine, Guangzhou, China; ^3^ The Ministry of Health, The First Affiliated Hospital of Guangzhou University of Chinese Medicine, Guangzhou, China; ^4^ National Malaria Control Programme, São Tomé and Príncipe, Guangzhou, China

**Keywords:** malaria, vector control, *Anopheles gambiae*, resistance, *kdr*, *ace^-1R^*

## Abstract

Malaria, one of the most serious parasitic diseases, kills thousands of people every year, especially in Africa. São Tomé and Príncipe are known to have stable transmission of malaria. Indoor residual spraying (IRS) of insecticides and long-lasting insecticidal nets (LLIN) are considered as an effective malaria control interventions in these places. The resistance status of *Anopheles gambiae s.s.* from Agua Grande, Caue, and Lemba of São Tomé and Príncipe to insecticides, such as dichlorodiphenyltrichloroethane (DDT) (4.0%), deltamethrin (0.05%), permethrin (0.75%), fenitrothion (1.0%), and malathion (5.0%), were tested according to the WHO standard protocol. DNA extraction, species identification, as well as *kdr* and *ace-1^R^* genotyping were done with the surviving and dead mosquitoes post testing. They showed resistance to cypermethrin with mortality rates ranging from 89.06% to 89.66%. Mosquitoes collected from Agua Grande, Caue, and Lemba displayed resistance to DDT and fenitrothion with mortality rates higher than 90%. No other species were detected in these study localities other than *Anopheles gambiae s.s*. The frequency of L1014F was high in the three investigated sites, which was detected for the first time in São Tomé and Príncipe. No *ace^-1R^* mutation was detected in all investigated sites. The high frequency of L1014F showed that *kdr* L1014F mutation might be related to insecticide resistance to *Anopheles gambiae s.s.* populations from São Tomé and Príncipe. Insecticide resistance status is alarming and, therefore, future malaria vector management should be seriously considered by the government of São Tomé and Príncipe.

## Introduction

Malaria, one of the most deadly parasitic diseases, has claimed thousands of people every year worldwide, especially in Africa. In 2019, nearly 229 million cases of malaria occurred worldwide with the high mortality (409,000), of which 80% occurred in sub-Saharan Africa. Malaria is thus a major limiting factor in the socio-economic development of Africa (WHO, 2018c). *Anopheles gambiae s.s.* was the only malaria vector found in São Tomé and Príncipe (Pinto et al., 2000). The country has a suitable climate and rich vegetation which is conducive for mosquito breeding and survival.

In São Tomé and Príncipe, malaria control strategies, such as long-lasting insecticidal nets (LLINs), indoor residual spraying (IRS) of insecticides, are carried out. Furthermore, intermittent preventive therapy treatment (IPT) during pregnancy, early diagnosis, and treatment using artemisinin-based combination therapy (ACT) have also been implemented (Lee et al., 2010b). Indoor residual spraying of insecticides, including dichlorodiphenyltrichloroethane (DDT), malathion, fenitrothion, deltamethrin, and cypermethrin, has been the anti-malarial intervention used. Cypermethrin was the most recommended compounds for IRS and LLINs in these late years as it is a fast-acting and low-toxicity insecticide (Graham et al., 2005). Malaria vector control dominated the anti-malaria measures in the last decade. IRS of insecticides was carried out for the 16th time in October 2019 in São Tomé and Príncipe. The evidence of malaria reduction coupled with IRS was reported in São Tomé and Príncipe (Hagmann et al., 2003; Lee et al., 2010b).

Since 2009, malaria incidence has decreased to less than 1% and morality rate fell to zero with the application of IRS using DDT and pyrethroid insecticides (Lee et al., 2010a). However, in 2018, 2978 malaria cases were reported by the National Malaria Control Programme of São Tomé and Príncipe. These cases increased in a larger extent from 2014 to 2017. Malaria positive rates were: 2.18% in Agua Grande, 1.86% in Lemba, 1.52% in Caue, 1.08% in Cantagalo, 1.01% in Me-Zochi, 0.54% in Lobata, and 0.39% in Príncipe from the database of the National Malaria Control Programme of São Tomé and Príncipe. However, no local malaria cases were found in some villages for more than three consecutive years compared with almost 30% of malaria cases in other villages.

Unfortunately, it was found that many malaria vectors are resistant to dichlorodiphenyltrichloroethane (DDT), deltamethrin, and cypermethrin used in public malaria interventions in Africa (WHO, 2018a). Out of which, resistance to pyrethroids and DDT has been reported in west and central Africa (Djogbenou et al., 2008; Kerah-Hinzoumbe et al., 2008; Moreno et al., 2008; Djegbe et al., 2011; Djogbenou et al., 2011; Namountougou et al., 2012; Aikpon et al., 2013; Nwane et al., 2013; Dery et al., 2016). In São Tomé and Príncipe, *Anopheles gambiae s.s.* mosquito mortality rates were 99% to 100% for carbamates from seven tested sites in 2014 to 2015, which meant *Anopheles gambiae s.s.* were still fully susceptible to carbamates. Target site of *kdr* L1014 had been detected with no reporting of testing for the *kdr* L1014 S in any species, and no *ace-1* ^R^ had been detected (WHO, 2018a).

The crucial mechanisms of insecticide resistance are enhanced detoxification and the voltage-gated sodium channel gene, known as knockdown resistance (*kdr*) and insensitive acetylcholinesterase (*ace-1*) gene (Vulule et al., 1999; Hemingway et al., 2004). The sodium channel gene at codon 1014 (1014F, 1014S) with two amino acid changes are related to *kdr* in *Anopheles gambiae s.s.* (Martinez-Torres et al., 1998; Ranson et al., 2000). Consequently, resistance to DDT and pyrethroids is caused by target site mutations (1014F and 1014S), whereas resistance to organophosphates and carbamates caused by *ace-1* results in a single amino acid substitution, changing the position 119, from glycine to serine (Weill et al., 2004).

The increase in malaria cases in São Tomé and Príncipe indicated that the long-term use of insecticides cannot eliminate malaria. This may be because of the acquired resistance in mosquitos to insecticides. Hence, it is necessary to carry out studies on insecticide resistance and its mechanism, which may help the government to control malaria. In this study, insecticide resistance, distribution, and frequency of *kdr* (L1014F, L1014S) mutation of *Anopheles gambiae s.s.* were performed in Agua Grande, Lemba, and Caue counties of São Tomé and Príncipe.

## Methods

### Study Sites

**Figure 1:**
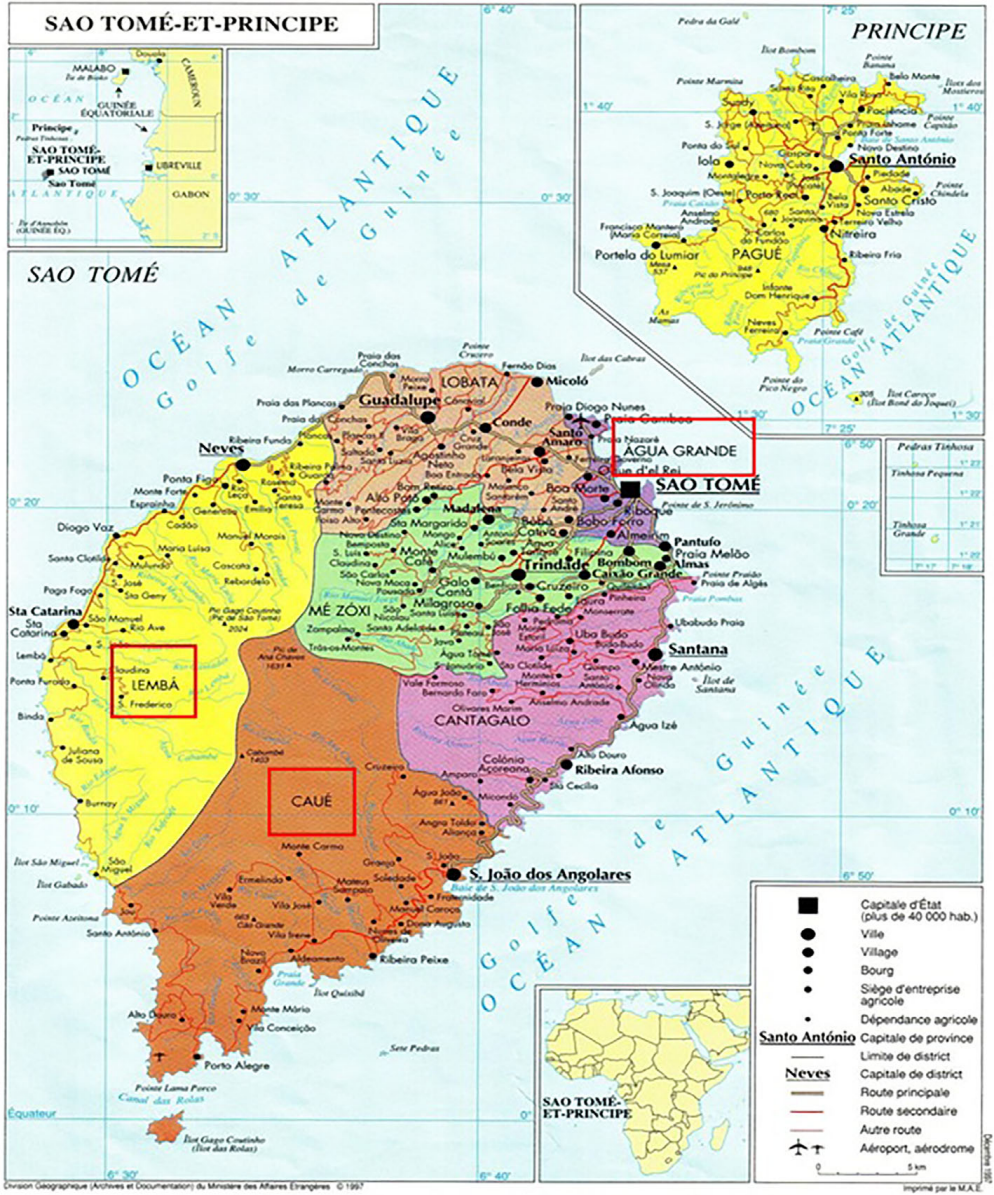
The map of São Tomé and Príncipe.

Three counties of São Tomé and Príncipe with high malaria positive rates were selected for this study (**Figure 1** and **Table 1**). São Tomé and Príncipe is an island country located near the equator, with a population of 200 thousand inhabitants. It has a tropical rainy climate of hot and humid condition all the year. It is characterized by rainy seasons (January to May and October to December) and one dry season (June to September) with a temperature ranging from 19°C to 30°C. The average rainfall ranges from 1000 to 2500 mm, whereas an average relative humidity ranges from 77% to 85% monthly. Moreover, the large planted surface area provided a favorable breeding condition for mosquitoes.

**Table 1:** Characteristics of study sites.

Localities	Geographic coordinates	Periods of collection
Agua Grande	N 0.33998 E 6.72446	September to December, 2018
Caue	N 0.06685 E 6.53714	September to December, 2018
Lemba	N 0.27986 E 6.50967	September to December, 2018

### Adult Mosquito Density Survey

Adult mosquitoes were captured using the human landing catch (HLC) technique in the study counties from 9:00 pm to 2:00 am by trained agents. At each capture site, two agents took turns during the night (each hour or between the first and the second part of the night). Each agent was equipped with a flashlight, hemolysis tubes, cotton, bags with notes on the capture point (indoor or outdoor), and the time intervals of the capture. An agent must capture the mosquito before the mosquito bites him after landing on his bare legs. In the dark, the agent turned on his flashlight as soon as he feels the mosquito landing on him and captured the mosquito before it bites the hemolysis tube and covers the tube with cotton. Once the tube clogged, the tube was introduced in the bag corresponding to the location and hour of capture. The capture was conducted at each site and at each passage in at least two capture points. At each site, the capture was done two times a week, inside the room/dwelling and outside (veranda). The collectors must rotate from 1 day to the next, depending on the location and the hours of capture. *Anopheles gambiae s.s.* were then counted after identification, and the mosquito density was calculated as “number of mosquitoes/collectors •40 h”.

### Larva Mosquito Density Survey

Mosquito larva density surveillance was conducted using the ladle method by specific people at specific time (from 8:00 am to 2:00 pm) and at specific mosquito breeding sites in each county. The surveying team was made up of two experienced technicians. The prospecting of mosquito breeding sites consists of, directly or when needed, taking water from the larval habitats with a ladle in order to search for larvae or nymphs. Water was taken by a ladle 15 times with 1 to 2 min between each take or 15 times with one time each 2-m distance. Take at least 1 L of water, count the number of larvae/nymphs. If the volume of water of the larval habitats is less than 1 L, calculate according to the following formula: the total number of larvae or nymphs/the volume of water (L). Larva density: number of collected larvae and nymphs/total number of take (with ladle).

### Mosquito Rearing

Instar larvae of *Anopheles gambiae s.l.* collected from larva density survey was pooled together by locality. The instar larvae of *Anopheles gambiae s.l*. were reared in the insectary of Centre for Disease Control and Prevention of São Tomé and Príncipe under standard conditions of 25 ± 2°C temperature and 80 ± 10% relative humidity (RH).

### Insecticide Susceptibility Assays

Three-day-old F_0_ female mosquitoes were used for insecticide susceptibility tests. Insecticide bioassays were carried out according to the WHO standard protocol (WHO, 2018b). Insecticides, including DDT (4%), deltamethrin (0.05%), permethrin (0.75%), fenitrothion (1.0%), and malathion (5.0%), were tested at 25 ± 2°C temperature and 70% to 80% relative humidity.

For this, insecticide papers were bought from the Centre for Disease Control and Prevention, China. Approximately 20 to 30 female mosquitoes without blood feeding were selected randomly for each batch, three batches were tested, and exposed to on impregnated paper for 1 h, and mosquitoes knockdown were counted every 10 min. Mosquitoes were then transferred into observation tubes under standard condition and provided with a pad of cotton wool soaked in 10% sugar water (WHO, 2018b). Mosquitoes mortality rates were estimated 24-h post-exposure. Test tubes without insecticide-impregnated papers were taken as a control. Later dead and surviving mosquitoes were transferred to labeled separate Eppendorf tubes and kept at −20°C.

The DNAs from dead and surviving mosquitoes were extracted according to the manufacturer’s protocol (TaKaRa, Japan, brought from China) in the laboratory of Centre for Disease Control and Prevention of São Tomé and Príncipe, and then were transferred to the laboratory of Guangzhou University of Chinese Medicine for species identification and resistance mechanism characterization.

### Species Identification, *kdr* L1014F, *kdr* L1014S, and *ace*-*1^R^* Genotyping

The PCR was performed to distinguish the species of *Anopheles gambiae* mosquitoes. The screening of *kdr* mutation, L1014F and L1014S by PCR was performed as described by Bass et al. (2007). For PCR, two common primers were used: kdr-F (5′-CATGATCTGCCAAGATGGAA-3′), kdr-R (5′-GTTGGTGCAGACAAGGATGA-3′) S6 region. The PCR amplification was performed for 35 cycles. It included the first pre-denaturation at 95°C for 3 min, then denaturation at 95°C for 30 s, annealing at 55°C for 30 s, and extension at 72°C for 30 s. Heated for 7 min at 72°C, the refrigerator was set at 4°C. The expected band size of 172 bp distinguished the resistant allele in the sibling species.

The presence of G119S-ace1 allele was screened as previously described (Bass et al., 2010) with primers of ace-1 F (5′-GATCGTGGACACCGTGTTCG-3′) and ace-1 R (5′-AGGATG GCCCGCTGGAACAG-3′). The PCR amplification was performed for 35 cycles. It included the first pre-denaturation at 95°C for 3 min, then denaturation at 95°C for 30 s, annealing at 55°C for 30 s, and extension at 72°C for 30 s. Heated for 7 min at 72°C, the refrigerator was set at 4°C. The band size of 541 bp was expected to distinguish between the resistant allele in the sibling species.

### Statistical Analysis

To check the resistance of mosquitoes according to the WHO criteria (WHO, 2018b): susceptibility of the mosquitoes is indicated for mortality rate in range of 98% to 100%.

Suspected resistance of the mosquitoes is indicated for mortality rate between 90% and 97%.

Confirmation of resistant mosquitoes is indicated for mortality rate less than 90%.

The mortality rates of the test samples were calculated by adding the number of dead mosquitoes across all exposure replicates and then expressing this as a percentage of the total number of exposed mosquitoes. The SPSS software version 24.0 was used to calculate the 95% CI mortality rates.

The genotype frequencies of *kdr* and *ace-1^R^* were compared between resistant and susceptible mosquitoes using Fisher exact test.

## Results

### Adult Mosquito Density

Mosquitoes were less abundant in the dry season (June to September), and the highest number of individuals was collected from January to May, corresponding to the rainy season, which provides suitable ecological environment for mosquito breeding.

As shown in **Table 2**, the mosquito density in Agua Grande was quite high, with a monthly value of 26.75 (outdoor) in May. The mosquito density in the study counties showed that the highest mosquito density from January to May and absolutely falling down from June to September.

**Table 2:** Adult mosquito density survey in the study sites in 2018.

Month	Adult mosquito density (number of mosquitoes/collectors 40 h)					
	Agua Grande		Lema		Caue	
	Indoor	Outdoor	Indoor	Outdoor	Indoor	Outdoor
January	0.125	6.125	0	0.750	0	4.125
February	0	0.125	0	0	0.375	2.875
March	0	2.375	0	0.875	1.125	9.875
April	0	9.000	0.500	5.750	1.000	6.750
May	0	26.750	0.125	3.125	1.125	5.250
June	0	5.250	0	4.000	1.500	12.750
July	0	1.375	0	4.500	0.750	4.500
August	0	3.875	0	3.375	0.125	5.500
September	0.125	1.875	1.125	6.625	0.375	3.125
October	0	10.000	0	3.870	0.280	2.280
November	0.250	8.870	0	2.420	0.500	3.250
December	0	17.600	2.000	2.250	0	3.160

### Larva Mosquito Density

The mosquito larva density was higher in the rainy season (January to May), that could provide suitable ecological environment for mosquito breeding. Few larvae were collected in the dry season (June to September).

As shown in **Table 3**, the mosquito larva density in Agua Grande was higher than other counties, with a monthly value of 4.90 in January and 6.96 in February. The mosquito larva density in the study counties showed that the highest larva density from January to May and absolutely falling down from June to September.

**Table 3:** Larva mosquito larva density survey in the study sites in 2018.

Month	Mosquito larva density (number of collected larvae and nymphs/total number of take)		
	Agua Grande	Lema	Caue
January	4.90	1.20	0.42
February	6.96	0.44	0.20
March	0.96	1.97	0.28
April	1.55	1.25	0
May	2.23	2.03	0
June	0.83	1.70	0
July	0	1.73	0
August	0	1.58	0
September	0.33	0.70	0
October	0.88	0.73	1.60
November	0.63	0.74	0.04
December	1.06	1.26	0.72

### Species Composition of *Anopheles gambiae s.l.*

The species identification was performed by PCR for the F_0_ females in *Anopheles gambiae* complex (n=1009) (**Table 5**). It was found that *Anopheles gambiae s.s.* (100%) was the only species of these three study sites.

### Insecticide Resistance Status

The insecticide resistance status of *An. gambiae s.s.* collected from different study sites were represented in **Table 4**. It was observed that the pyrethroid*-*resistant *Anopheles gambiae s.s.* were found in the three study sites with the highest mortality rates were 89.66% [CI (89.54 ± 1.34)] for 0.75% Cypermethrin and 88.57% [CI (89.17 ± 7.40)] for 0.05% Deltamethrin in Lemba. The *Anopheles gambiae s.s.*, resistant to 5% Malathion [89.71%, CI (89.65 ± 2.54)] was also observed in Caue. The mosquitoes were suspected resistant to 4% DDT and 1% Fenitrothion.

**Table 4:** Susceptibility status of *Anopheles gambiae s.s.* collected in the study sites and exposed to the five classes of insecticides.

Insecticides	Study sites	Species	N	Mortality (%)	95%CI	Susceptibility status
4%DDT	Agua Grande	*Anopheles gambiae s.s.*	56	92.86	93.01 ± 2.20	Suspected resistant
	Caue	*Anopheles gambiae s.s.*	60	93.33	93.24 ± 3.26	Suspected resistant
	Lemba	*Anopheles gambiae s.s.*	69	92.75	92.89 ± 1.84	Suspected resistant
0.05% Deltamethrin	Agua Grande	*Anopheles gambiae s.s.*	82	91.46	91.23 ± 3.24	Suspected resistant
	Caue	*Anopheles gambiae s.s.*	78	84.62	85.11 ± 9.58	Resistant
	Lemba	*Anopheles gambiae s.s.*	70	88.57	89.17 ± 7.40	Resistant
0.75% Cypermethrin	Agua Grande	*Anopheles gambiae s.s.*	64	89.06	89.05 ± 2.18	Resistant
	Caue	*Anopheles gambiae s.s.*	57	89.47	89.63 ± 4.18	Resistant
	Lemba	*Anopheles gambiae s.s.*	58	89.66	89.54 ± 1.34	Resistant
1% Fenitrothion	Agua Grande	*Anopheles gambiae s.s.*	73	90.41	90.84 ± 5.01	Suspected resistant
	Caue	*Anopheles gambiae s.s.*	75	92.00	92.35 ± 2.37	Suspected resistant
	Lemba	*Anopheles gambiae s.s.*	66	90.91	91.21 ± 3.11	Suspected resistant
5% Malathion	Agua Grande	*Anopheles gambiae s.s.*	61	91.80	91.92 ± 2.28	Suspected resistant
	Caue	*Anopheles gambiae s.s.*	68	89.71	89.65 ± 2.54	Resistant
	Lemba	*Anopheles gambiae s.s.*	72	93.06	93.18 ± 1.79	Suspected resistant

N, number of mosquitoes; Cl, confidence interval.

### Detection of Resistance Genes

DNA was extracted from dead and surviving mosquitoes of insecticide susceptibility assays in every study site for checking *kdr* and *ace-1^R^*. The *kdr* mutation of *Anopheles gambiae s.s.* only detected one heterozygous mutations type (TTA/TTT): leucine to phenylalanine substitution (L1014F). The mutation frequency of *kdr* (L1014F) and *ace-1^R^* (G119S) is shown in **Table 5**. The L1014F *kdr* mutation was observed in *Anopheles gambiae s.s.* in all the three study sites with frequencies ranging from 8.33% to 43.75%, and it appeared for the first time in São Tomé and Príncipe. However, no *ace-1^R^* mutation was detected in *Anopheles gambiae s.s.* in all the three study sites.

**Table 5:** Distribution and Resistant allele frequencies in *Anopheles gambiae s.s. in* study sites from São Tomé and Príncipe.

Insecticides	Study sites	Species	Bioassay*	N	*kdr* Genotypes		f(L1014F)	*p*	*ace^-1^* Genotypes		G119S%
					TTA/TTA	TTA/TTT			GGC/GGC	GGC/AGC	
0.75% Cypermethrin	Agua Grande	*Anopheles gambiae s.s.*	Resistant	24	3	21	0.4375	0.000^△^	–	–	–
			Susceptible	24	24	0	0		–	–	–
	Caue	*Anopheles gambiae s.s.*	Resistant	24	20	4	0.0833	0.109^△^	–	–	–
			Susceptible	24	24	0	0		–	–	–
	Lemba	*Anopheles gambiae s.s.*	Resistant	24	9	15	0.3125	0.000^△^	–	–	–
			Susceptible	24	24	0	0		–	–	–
5% Malathion	Agua Grande	*Anopheles gambiae s.s.*	Resistant	24	–	–	–		24	0	0
			Susceptible	24	–	–	–		24	0	0
	Caue	*Anopheles gambiae s.s.*	Resistant	24	–	–	–		24	0	0
			Susceptible	24	–	–	–		24	0	0
	Lemba	*Anopheles gambiae s.s.*	Resistant	24	–	–	–		24	0	0
			Susceptible	24	–	–	–		24	0	0

*”Resistant” refers to the mosquitoes that were alive 24 h after 1-h exposure to the insecticides in the standard WHO tube bioassay; and “susceptible” refers to the mosquitoes that were knocked down within the 24-h recovery period. ^△^Fisher Exact test.

## Discussion

The study showed that the levels of insecticide resistance in *Anopheles gambiae s.s.* were high in São Tomé and Príncipe with high allelic frequencies. *Anopheles gambiae s.s.* was resistant to DDT and pyrethroids with the allele targeting the voltage-gate sodium channel. Only one species of malaria vector, i.e., *Anopheles gambiae s.s.*, was identified in this study. In this species, *kdr* allele frequencies were high at Agua Grande and Lemba, and for the first time, the high frequency *kdr* mutation of L1014F was detected in São Tomé and Príncipe.

Current status of pyrethroid resistance in *Anopheles gambiae s.s.* can also be provided to the National Malaria Control Programme of São Tomé and Príncipe from the study. The insecticide susceptibility assay results demonstrated a high level of DDT and pyrethroids resistance at study sites. It also suggested that there are many selection pressures on choosing these insecticides. Clément Kerah-Hinzoumbé et al. reported the resistance to permethrin and deltamethrin with varying concentration, and the *kdr* mutation of L1014F appeared as the S form of *Anopheles gambiae* in Chad (Kerah-Hinzoumbe et al., 2008). In Cameroon, the incidence of DDT resistance and the heterogenous levels of susceptibility to deltamethrin and permethrin were extensive in *Anopheles gambiae s.s*, it was demonstrated that, the multiple resistance mechanisms segregate in *Anopheles gambiae* which resulted in the heterogeneous resistance profiles (Nwane et al., 2013). In Cameroon, the insecticide resistance due to DDT, bendiocarb, permethrin, and deltamethrin spread widely in *Anopheles gambiae (s.l.)*. From 2000 to 2017, the prevalence of *kdr* allele frequency increased steadily in all study sites in *Anopheles gambiae (s.l.)*, with the L1014F *kdr* allele frequency was the most extensive (Antonio-Nkondjio et al., 2017). Furthermore, it was reported that there was some resistance to the insecticides such as pyrethroids and DDT in the Kassena-Nankana district of Ghana in 2009 (Anto et al., 2009). In this study, the results suggested that the increase of pyrethroid resistance was greatly enhanced during last 3 years, which may be due to the selection pressure, such as mass implementation of IRS and mass distribution of LLINs. The continuous application of IRS with the pyrethroid insecticides resulted in the increase of the resistance. Therefore, we observed high frequencies of *kdr* alleles in these study sites. The *kdr* allele dominated in the mechanism of resistance to DDT and pyrethroids in experimental mosquitos.

The resistance of pyrethroid is worrying, and as per our knowledge, it can influence the current vector intervention strategies, such as pyrethroid-only in IRS and LLINs. This target site of *kdr* mutation could be the reason of high resistance to deltamethrin and permethrin in *Anopheles gambiae s.s.* in São Tomé and Príncipe. Further, the L1014S allele related to *Anopheles gambiae s.s.* resistance appeared in Congo in 2013 (Basilua Kanza et al., 2013). Similarly, the pyrethroid resistance related to *kdr* L1014F and *ace* ^-1R^ G119S mutation was first reported in Togo (Djegbe et al., 2018).

Here, we found that most of the malaria cases were in Agua Grande with the lowest coverage rate 64.55% of IRS, which indicated that *Anopheles gambiae s.s.* in Agua Grande may be resistant to insecticides used in IRS. From the results of this study, it is necessary to pay attention for the use of insecticides and urge the decision makers (National Malaria Control Programme of São Tomé and Príncipe) for an urgent change of resistance management program.

As we all know, study on the mechanism of Pyrethroids resistance of mosquitoes include two categories: metabolic detoxification and reduced sensitivity of the target.

Other metabolic resistance mechanisms (detoxification genes) particularly P450 monooxygenase and GST, which are primarily associated with DDT, pyrethroids, and bendiocarb resistance are really important. As the lack of the special equipments and reagents for metabolic resistance mechanisms, especially for Sao Tome and Principe, a Low-Middle Income Country, we could not monitor the metabolic resistance mechanisms in the study. However, special equipments and reagents for metabolic resistance mechanisms will be considered to bring into São Tomé and Príncipe for the future work. We found that more research should be needed and designed more rigorously to study the resistance mechanism of mosquitoes and serious intervention malaria programs in São Tomé and Príncipe.

## Conclusion

This study showed that *Anopheles gambiae s.s.* in São Tomé and Príncipe was resistant to almost all the investigated insecticides. The increase in malaria cases in these years may be related to the resistance allele of L1014F(TTA/TTT) that was identified, representing a serious threat. Therefore, it is necessary to devise a malaria vector control strategy in Sao Tome and Principe urgently. The combined role of the mosquito resistance mechanism provides a scientific basis for local mosquito vector interventions in São Tomé and Príncipe.

## Data Availability Statement

The original contributions presented in the study are included in the article/supplementary material. Further inquiries can be directed to the corresponding author.

## Author Contributions

QW and JS designed, organized, and supervised the program. HZ and QW designed the study protocol, analyzed data, and wrote the manuscript. ML, RT, SZ, FT, WG, CB, and DR helped carry out the field work. BH and YY performed molecular and biochemical analyses. CD and QX revised the manuscript. All authors contributed to the article and approved the submitted version.

## Funding

This work was supported by Natural Science Foundation of China [Grant Number 81873218] to JS and QW, Natural Science Foundation of China [Grant Number 82074301] to CD, Guangdong Provincial Science and Technology Plan Project [Grant Number 2020A0505090009] to QW and YY, Guangdong Provincial Science and Technology Plan Project [Grant Number 2021A0505030060] to JS, and Project of Traditional Chinese Medicine Bureau [Grant Number GZYYGJ2020030] to JS and CD. The funders had no role in study design, data collection and analysis, decision to publish, or preparation of the manuscript.

## Conflict of Interest

The authors declare that the research was conducted in the absence of any commercial or financial relationships that could be construed as a potential conflict of interest.

## References

[B1] Aikpon R. Agossa F. Osse R. Oussou O. Aizoun N. Oke-Agbo F.. (2013). Bendiocarb Resistance in Anopheles Gambiae s.l. Populations From Atacora Department in Benin, West Africa: A Threat for Malaria Vector Control. Parasit. Vectors 6, 192. 10.1186/1756-3305-6-192 23803527 PMC3698110

[B2] Anto F. Asoala V. Anyorigiya T. Oduro A. Adjuik M. Owusu-Agyei S.. (2009). Insecticide Resistance Profiles for Malaria Vectors in the Kassena-Nankana District of Ghana. Malar. J. 8, 81. 10.1186/1475-2875-8-81 19389257 PMC2685403

[B3] Antonio-Nkondjio C. Sonhafouo-Chiana N. Ngadjeu C. S. Doumbe-Belisse P. Talipouo A. Djamouko-Djonkam L.. (2017). Review of the Evolution of Insecticide Resistance in Main Malaria Vectors in Cameroon From 1990 to 2017. Parasit. Vectors 10, 472. 10.1186/s13071-017-2417-9 29017590 PMC5635606

[B4] Basilua Kanza J. P. El Fahime E. Alaoui S. Essassi El M. Brooke B. Nkebolo Malafu A.. (2013). Pyrethroid, DDT and Malathion Resistance in the Malaria Vector Anopheles Gambiae From the Democratic Republic of Congo. Trans. R. Soc. Trop. Med. Hyg. 107, 8–14. 10.1093/trstmh/trs002 23222943

[B5] Bass C. Nikou D. Donnelly M. J. Williamson M. S. Ranson H. Ball A.. (2007). Detection of Knockdown Resistance (Kdr) Mutations in Anopheles Gambiae: A Comparison of Two New High-Throughput Assays With Existing Methods. Malaria J. 6, 111. 10.1186/1475-2875-6-111 PMC1971715 17697325

[B6] Bass C. Nikou D. Vontas J. Williamson M. S. Field L. M. (2010). Development of High-Throughput Real-Time PCR Assays for the Identification of Insensitive Acetylcholinesterase (Ace-1(R)) in Anopheles Gambiae. Pestic. Biochem. Physiol. 96, 80–85. 10.1016/j.pestbp.2009.09.004

[B7] Dery D. B. Segbaya S. Asoalla V. Amoyaw F. Amoako N. Agyeman-Budu A.. (2016). Anopheles Gambiae (Diptera: Culicidae) Susceptibility to Insecticides and Knockdown Resistance Genes Prior to Introduction of Indoor Residual Spraying in 11 Districts in Ghana. J. Med. Entomol. 53, 1403–1409. 10.1093/jme/tjw098 27330096

[B8] Djegbe I. Akoton R. Tchigossou G. Ahadji-Dabla K. M. Atoyebi S. M. Adeoti R.. (2018). First Report of the Presence of L1014S Knockdown-Resistance Mutation in Anopheles Gambiae s.s and Anopheles Coluzzii From Togo, West Africa. Wellcome Open Res. 3, 30. 10.12688/wellcomeopenres.13888.1 29707654 PMC5909049

[B9] Djegbe I. Boussari O. Sidick A. Martin T. Ranson H. Chandre F.. (2011). Dynamics of Insecticide Resistance in Malaria Vectors in Benin: First Evidence of the Presence of L1014S Kdr Mutation in Anopheles Gambiae From West Africa. Malaria J. 10, 261. 10.1186/1475-2875-10-261 PMC3179749 21910856

[B10] Djogbenou L. Dabire R. Diabate A. Kengne P. Akogbeto M. Hougard J. M.. (2008). Identification and Geographic Distribution of the ACE-1R Mutation in the Malaria Vector Anopheles Gambiae in South-Western Burkina Faso, West Africa. Am. J. Trop. Med. Hyg. 78, 298–302. 10.4269/ajtmh.2008.78.298 18256433

[B11] Djogbenou L. Pasteur N. Akogbeto M. Weill M. Chandre F. (2011). Insecticide Resistance in the Anopheles Gambiae Complex in Benin: A Nationwide Survey. Med. Vet. Entomol. 25, 256–267. 10.1111/j.1365-2915.2010.00925.x 21155858

[B12] Graham K. Kayedi M. H. Maxwell C. Kaur H. Rehman H. Malima R.. (2005). Multi-Country Field Trials Comparing Wash-Resistance of PermaNet and Conventional Insecticide-Treated Nets Against Anopheline and Culicine Mosquitoes. Med. Vet. Entomol. 19, 72–83. 10.1111/j.0269-283X.2005.00543.x 15752180

[B13] Hagmann R. Charlwood J. D. Gil V. Ferreira C. Do Rosario V. Smith T. A. (2003). Malaria and its Possible Control on the Island of Principe. Malaria J. 2, 15. 10.1186/1475-2875-2-15 PMC166171 12875660

[B14] Hemingway J. Hawkes N. J. Mccarroll L. Ranson H. (2004). The Molecular Basis of Insecticide Resistance in Mosquitoes. Insect Biochem. Mol. Biol. 34, 653–665. 10.1016/j.ibmb.2004.03.018 15242706

[B15] Kerah-Hinzoumbe C. Peka M. Nwane P. Donan-Gouni I. Etang J. Same-Ekobo A.. (2008). Insecticide Resistance in Anopheles Gambiae From South-Western Chad, Central Africa. Malar. J. 7, 192. 10.1186/1475-2875-7-192 18823537 PMC2566574

[B16] Lee P. W. Liu C. T. Do Rosario V. E. De Sousa B. Rampao H. S. Shaio M. F. (2010a). Potential Threat of Malaria Epidemics in a Low Transmission Area, as Exemplified by Sao Tome and Principe. Malar. J. 9, 264. 10.1186/1475-2875-9-264 20920216 PMC2955676

[B17] Lee P. W. Liu C. T. Rampao H. S. Do Rosario V. E. Shaio M. F. (2010b). Pre-Elimination of Malaria on the Island of Principe. Malar. J. 9, 26. 10.1186/1475-2875-9-26 20089158 PMC2823607

[B18] Martinez-Torres D. Chandre F. Williamson M. S. Darriet F. Berge J. B. Devonshire A. L.. (1998). Molecular Characterization of Pyrethroid Knockdown Resistance (Kdr) in the Major Malaria Vector Anopheles Gambiae S.S. Insect Mol. Biol. 7, 179–184. 10.1046/j.1365-2583.1998.72062.x 9535162

[B19] Moreno M. Vicente J. L. Cano J. Berzosa P. J. De Lucio A. Nzambo S.. (2008). Knockdown Resistance Mutations (Kdr) and Insecticide Susceptibility to DDT and Pyrethroids in Anopheles Gambiae From Equatorial Guinea. Trop. Med. Int. Health 13, 430–433. 10.1111/j.1365-3156.2008.02010.x 18397404

[B20] Namountougou M. Simard F. Baldet T. Diabate A. Ouedraogo J. B. Martin T.. (2012). Multiple Insecticide Resistance in Anopheles Gambiae s.l. Populations From Burkina Faso, West Africa. PloS One 7 (11), e48412. 10.1371/journal.pone.0048412 23189131 PMC3506617

[B21] Nwane P. Etang J. Chouasmall Yi U. M. Toto J. C. Koffi A. Mimpfoundi R.. (2013). Multiple Insecticide Resistance Mechanisms in Anopheles Gambiae s.l. Populations From Cameroon, Central Africa. Parasit. Vectors 6, 41. 10.1186/1756-3305-6-41 23433176 PMC3583743

[B22] Pinto J. Sousa C. A. Gil V. Ferreira C. Goncalves L. Lopes D.. (2000). Malaria in Sao Tome and Principe: Parasite Prevalences and Vector Densities. Acta Trop. 76, 185–193. 10.1016/S0001-706X(00)00100-5 10936578

[B23] Ranson H. Jensen B. Vulule J. M. Wang X. Hemingway J. Collins F. H. (2000). Identification of a Point Mutation in the Voltage-Gated Sodium Channel Gene of Kenyan Anopheles Gambiae Associated With Resistance to DDT and Pyrethroids. Insect Mol. Biol. 9, 491–497. 10.1046/j.1365-2583.2000.00209.x 11029667

[B24] Vulule J. M. Beach R. F. Atieli F. K. Mcallister J. C. Brogdon W. G. Roberts J. M.. (1999). Elevated Oxidase and Esterase Levels Associated With Permethrin Tolerance in Anopheles Gambiae From Kenyan Villages Using Permethrin-Impregnated Nets. Med. Vet. Entomol. 13, 239–244. 10.1046/j.1365-2915.1999.00177.x 10514048

[B25] Weill M. Malcolm C. Chandre F. Mogensen K. Berthomieu A. Marquine M.. (2004). The Unique Mutation in Ace-1 Giving High Insecticide Resistance Is Easily Detectable in Mosquito Vectors. Insect Mol. Biol. 13, 1–7. 10.1111/j.1365-2583.2004.00452.x 14728661

[B26] Who. (2018a). Global Report on Insecticide Resistance in Malaria Vectors: 2010-2016. (Geneva: World Health Organization)

[B27] Who. (2018b). Test Procedures for Insecticide Resistance Monitoring in Malaria Vector Mosquitoes, 2nd ed. (Geneva: World Health Organization).

[B28] Who. (2018c). World Malaria Report 2018. (Geneva: World Health Organization).

